# Fra-1/AP-1 Transcription Factor Negatively Regulates Pulmonary Fibrosis *In Vivo*

**DOI:** 10.1371/journal.pone.0041611

**Published:** 2012-07-24

**Authors:** Subbiah Rajasekaran, Michelle Vaz, Sekhar P. Reddy

**Affiliations:** 1 Department of Pediatrics, University of Illinois at Chicago, Chicago, Illinois, United States of America; 2 Department of Environmental Health Sciences, Bloomberg School of Public Health, The Johns Hopkins University, Baltimore, Maryland, United States of America; Johns Hopkins School of Medicine, United States of America

## Abstract

The Fra-1/AP-1 transcription factor plays a key role in tumor epithelial cell progression; however, its role in pathogenic lung fibrosis remains unclear. In the present study, using a genetic approach (Fra-1 deficient mice), we have demonstrated a novel regulatory (protective) role for Fra-1 in lung fibrosis. We found greater levels of progressive interstitial fibrosis, characterized by increased levels of inflammation, collagen accumulation, and profibrotic and fibrotic gene expression in the lungs of *Fra-1* ^Δ/Δ^ mice than in those of *Fra-1^+/+^* mice following bleomycin treatment. Fra-1 knockdown in human lung epithelial cells caused the upregulation of mesenchymal marker N-cadherin, concomitant with a downregulation of the epithelial phenotype marker E-cadherin, under basal conditions and in response to bleomycin and TGF-β1. Furthermore, Fra-1 knockdown caused an enhanced expression of type 1 collagen and the downregulation of collagenase (MMP-1 and MMP-13) gene expression in human lung epithelial cells. Collectively, our findings demonstrate that Fra-1 mediates anti-fibrotic effects in the lung through the modulation of proinflammatory, profibrotic and fibrotic gene expression, and suggests that the Fra-1 transcription factor may be a potential target for pulmonary fibrosis, a progressive disorder with poor prognosis and treatment.

## Introduction

Pulmonary fibrosis is a chronic, progressive disorder that leads to morbidity and mortality and is associated with poor prognosis and treatment. This disease is characterized by fibroblast proliferation, extracellular matrix (ECM) accumulation, and alterations in parenchymal architecture leading to scar formation [1], but the exact mechanisms underlying this pathogenic fibrosis are not completely defined. Increased production of interstitial collagens as a result of ECM remodeling and protease and antiprotease imbalance has been implicated in both experimental (bleomycin-induced) [2] and human pulmonary fibrosis [3]. The matrix metalloproteinase (MMP) family members regulate ECM turnover, whereas collagenases (members of MMP subfamily) cleave the interstitial collagens, types I, II and III under both physiologic and pathologic conditions. The activities of these enzymes are controlled at multiple levels, in particular through the interactions with their specific inhibitors, known as the tissue inhibitors of metalloproteinases (TIMPs). Thus, a concerted regulation of both MMP and TIMP expression is critical to maintaining tissue homeostasis and remodeling during normal physiological processes such as development and wound healing [4]. However, the loss of this coordinated regulation of MMP and TIMP expression has been shown to contributes to the development and progression of several diseases, including fibrosis [5], [6], [7].

The AP-1 transcription factor, mainly comprised of the Jun (c-Jun, Jun-B, Jun-D) and Fos (c-Fos, Fos-B, Fra-1, Fra-2) families of b-ZIP transcription factors, binds to the TPA response element (TRE, also known as the AP-1 site) of target gene promoters and regulates their expression in response to various pro-oxidants and toxicants. These gene products mediate (mitigate or promote) oxidative stress and inflammatory responses, as well as cell growth and tumorigenesis [8]. These diverse cellular processes mediated by AP-1 family members in response to various physiological and pathogenic stimuli have generally been attributed to the nature of activation of Jun and Fos family members, their dimeric composition, and the duration of the subsequent TRE-mediated induction of genes [9], [10]. Many growth factors and inflammatory cytokines implicated in lung fibrosis, including the TGF-β1, are known regulators of the AP-1 activity both *in vitro* and *in vivo* [10], [11]; however, the exact relevance of Jun and Fos family member activation in pro-fibrotic stimuli and their contribution to lung fibrosis are largely undefined. Several studies, including ours, have shown that ectopic Fra-1 expression upregulates the expression of genes controlling tissue/cell remodeling, such as MMP-1, MMP-2, and MMP-9, mainly at the transcriptional level [12], [13], [14], [15], [16]. Thus, we hypothesized that the Fra-1 transcription factor is critical for promoting lung fibrosis and mice lacking Fra-1 would not develop lung fibrosis *in vivo*. Here we report that, contrary to our expectation, deletion of Fra-1 led to an increased severity of lung fibrosis in an experimental model of bleomycin-induced lung injury. Furthermore, we found that the anti-fibrotic effects mediated by Fra-1 occur through the modulation of expression of pro-inflammatory, pro-fibrotic and fibrotic genes both *in vivo* and *in vitro*.

## Methods

### Mice

Conventional deletion of *Fra-1* resulted in embryonic lethality as a result of extra-embryonic tissue defects [17]. Therefore, in order to examine the role of this transcription factor in pulmonary fibrosis, the mice bearing a *Fra-1* “floxed” allele [17] (hereafter noated as Fra-1^FF^ mice) were obtained from Erwin F. Wagner (Ludwig Boltzmann Institute for Cancer Research, Vienna, Austria). These mice are maintained in a mixed (C57BL6/129) background. Meox2 (Sox2)-Cre transgenic mice (C57BL6/129), in which Cre expression specifically restricted in embryo but not in extra-embryonic tissues [18], were obtained from the Jackson Labs. Meox2 Cre mice were crossed to Fra-1^F/F^ mice , and the Fra-1^ Wt/F^-Meox2-Cre were then backcrossed to parent Fra-1^F/F^ mice to obtain Fra-1^F/F^-Meox2-Cre mice and the pups were genotyped for the lack of two “floxed” alleles of Fra-1 (Fra-1^F/F^) and the presence of Cre (data not shown). Fra-1^FF^ mice with and without Cre are hereafter referred to as *Fra-1* ^Δ/Δ^ and *Fra-1^+/+^* genotypes, respectively.

### Bleomycin Treatment

Bleomycin (0.075U) (APP Pharmaceuticals, LLC, Schaumburg, IL, USA) diluted in 30 µL of PBS was intratracheally administered to mice (10–14 weeks old) as described previously [19]. All experiments were conducted under a protocol approved by the institutional animal care use committee of the Johns Hopkins University and the University of Illinois at Chicago. At the end of experimental period, the right lobes were used for the bronchoalveolar lavage (BAL) collection and for the measurement of hydroxyproline content. The left lungs were fixed in 10% formalin for histological examination or were frozen immediately for subsequent gene expression analysis. The number of animals used for each analysis is given in figure legends.

### Measurement of Lung Inflammation, Fibrosis and Hydroxyproline Content

Inflammatory response in the lung tissue was analyzed by differential cell count and H&E staining. Fibrosis was detected by conventional trichrome staining of lung sections. Hydroxyproline content was measured by spectrophotometrically to quantify lung collagen accumulation as detailed elsewhere [20]. Briefly, lung tissues were washed in PBS, weighed, minced and diluted in 1 mL of PBS. Samples were then hydrolyzed in 12 N HCl at 110°C for overnight. Each sample (5 µL) was mixed with equal volume of citrate-acetate buffer and 100 µLs of chloramines-T solution. The mixture was incubated for 20 minutes at room temperature. Ehrlich’s solution was then added and the samples were incubated at 65°C for 18 minutes and absorbance was measured at 550 nm using hydroxyproline as a standard. The results were expressed as µg of hydroxyproline content/mg of lung tissue.

### Human Lung Epithelial Cell Culture for Small Interfering RNA (siRNA) Transfection

The human normal small airway epithelial cells (hereafter referred to as AECs) immortalized by telomerase [21] (kindly provided by Tom K. Hei and Chang Q. Piao, Columbia University, NY, USA), and A549 cells (obtained from ATCC) were cultured in DMEM/F12 and RPMI 1640 medium, respectively, supplemented with 10% FBS, 1% antibiotics, fungizone and growth factors. To silence *Fra-1* expression, AECs and A549 cells at 30–40% confluence were transfected with SMART pool siRNA specific for human Fra-1 or scrambled siRNA (Dharmacon) at 20 nM concentrations using DharmaFECT 1 transfection reagent (T-2001-01). After 48 hours incubation, cells were treated with bleomycin (5 mU/mL) or TGF-β1 (5 ng/mL) for different time points, total RNA and proteins were isolated.

### Real-time RT-PCR Analysis

Total RNA was extracted from lung tissues, AECs and A549 cells using TRIZOL reagent by following the manufacturer’s instructions (Invitrogen, CA, USA). For real-time RT-PCR analysis, 1 µg of total RNA was reverse transcribed using qscript cDNA supermix kit (Quanta Biosciences Inc., MD, USA). The expression levels of various genes were quantified in triplicate by TaqMan gene expression assays using β-actin as reference. The absolute expression value for each gene was normalized to that of β-actin and the values from control samples were set as one unit. Each experiment was repeated and *n* of at least 3 was derived from two independent experiments.

### Immunoblot Analysis

The whole protein lysate isolated from lung tissues and AECs was separated on a 10% SDS-PAGE and transferred to nitrocellulose membranes. Membranes were probed with primary antibodies against E-cadherin, N-cadherin (Santa Cruz Biotechnology Inc., CA, USA), type 1 collagen (Abcam Inc., Cambridge, MA, USA; Calbiochem, USA), Fra-1 (Santa Cruz Biotechnology Inc., CA, USA), β-actin, and α-tubulin (Sigma Aldrich, St. Louis, MO, USA). Immunoreactive bands were detected with appropriate horseradish peroxidase-conjugated secondary antibodies and visualized by enhanced chemiluminescence detection reagent.

### ELISA

Levels of active TGF-β1 in the lung tissue lysate were measured with an ELISA kit as per the manufacturer’s instructions (eBioscience Inc., USA).

### Collagen Zymographic Analysis

Because some MMPs can degrade type 1 collagen, zymographic techniques were used to detect their activity in tissues. Equal amount of protein extracted from lung tissues were separated on 10% SDS-PAGE gel containing 1 mg/ml of type 1 collagen prepared from the rat tail tendon (Invitrogen, USA). Gels were washed twice with 2.5% Triton X-100, rinsed with water, incubated overnight at 37°C in developing buffer (50 mM Tris, 5 mM CaCl_2,_ 2 µM ZnCl_2_, pH = 8) and stained with Coomasie brilliant blue in a solution containing 25% ethanol and 10% acetic acid. The enzyme activity appeared as clear bands against blue background. We used recombinant protein molecular weight markers to estimate the molecular weights of the proteolytic bands.

### Statistical Analysis

All data involving animal experimentation and cell culture studies were collected by an investigator or technician blinded to the specific experimental group used. Data were expressed as the mean ± SD. Student’s T test was used and the p value lower than 0.05 was considered significant.

## Results

### Fra-1 Deletion Results in Increased Levels of Lung Inflammation and Pro-inflammatory Cytokine Expression at Day 7 of Post-bleomycin Administration

The bleomycin-induced lung injury model is commonly used as an experimental model to elucidate the mechanisms underlying the development and progression of lung fibrosis. The development of fibrosis in this model can be seen biochemically and histologically by day 14 with maximal responses generally noted around 21 days [22], [23], [24], and begins to resolve after this period [25], [26], [27]. While the resolving nature of the fibrosis induced by bleomycin does not mimic human disease, this aspect of the model system offers an opportunity to study fibrotic resolution at these later time points. Thus, to determine whether Fra-1 regulates pulmonary fibrosis *in vivo*, *Fra-1* ^Δ/Δ^ and *Fra-1^+/+^* littermates were treated with vehicle or bleomycin, and the early and late fibrotic responses, histological changes, inflammatory cell profiles and proinflammatory cytokines and chemokines associated with this injury were evaluated at 7, 14, and 31 days after bleomycin administration.

**Figure 1:**
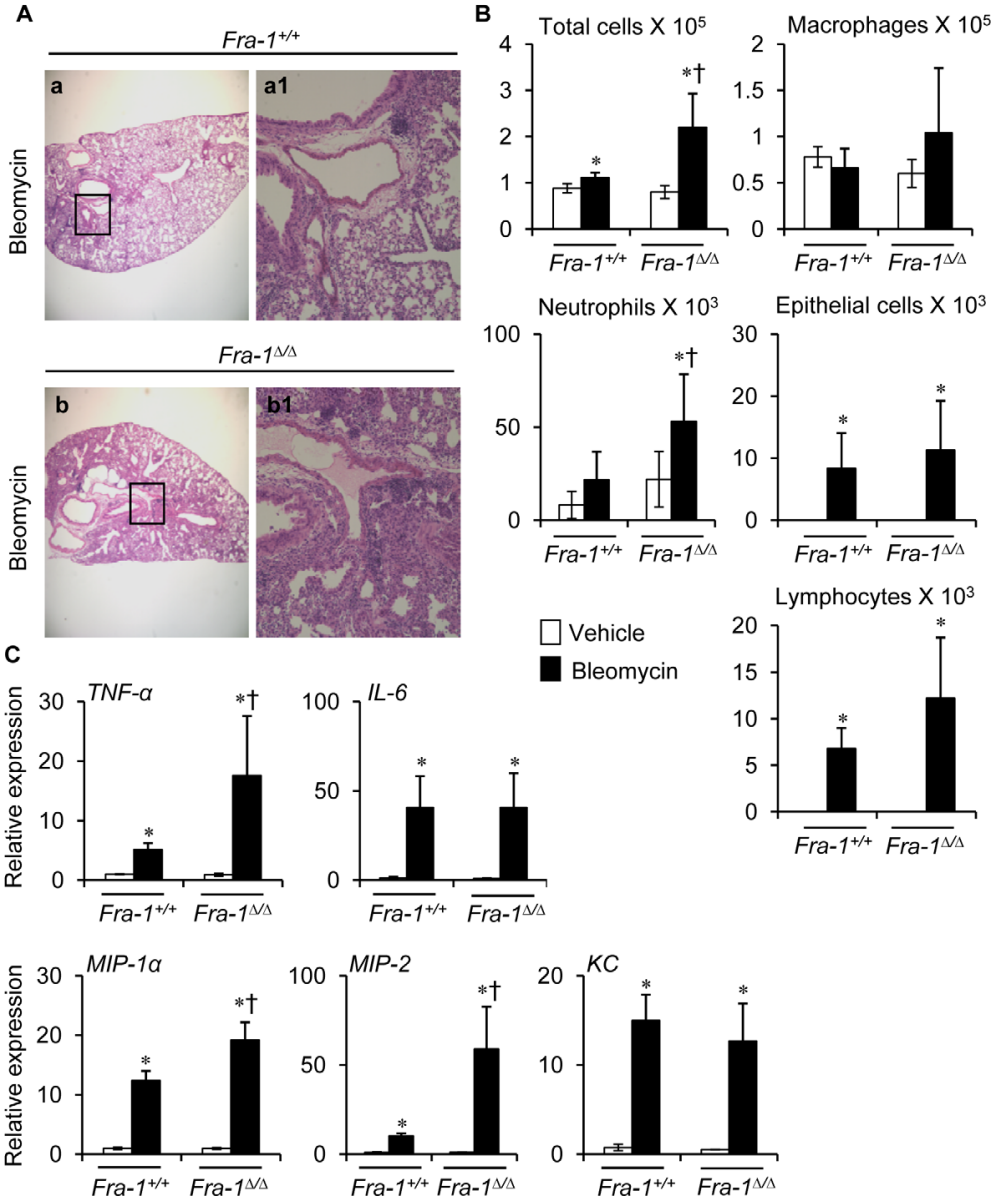
*Fra-1* ^Δ*/*Δ^ mice display greater pulmonary inflammatory responses at 7-day post-bleomycin administration. Mice (n = 8 in each genotype) were intratracheally administered with bleomycin. Left lungs were fixed for histology (n = 4) or used for mRNA analysis (n = 4). Right lobes (n = 8) were used for BAL and protein analysis. Representative results of two different experiments are shown. Samples obatined from mice treated with PBS were from Figure 3 (see below). **A:** Representative images of H&E stained lung tissue sections of bleomycin-treated *Fra-1^+/+^* and *Fra-1* ^Δ*/*Δ^ mice (n = 4). **B:** Assessment of lung inflammatory cell profiles in the BAL fluid of both genotypes (n = 5). **C:** Pro-inflammatory cytokines and chemokines gene expression after treatment with bleomycin (n = 4).^∗^p<0.05, PBS vs bleomycin; ^†^p*<*0.05, *Fra-1* ^Δ*/*Δ^ vs *Fra-1^+/+^* mice. **a** and **b** images shown at 4× magnification, while **a1** and **b1** are boxed areas of **a** and **b**, respectively, but shown at x20.

Histologic analyses at 7 days after bleomycin instillation revealed that *Fra-1* ^Δ/Δ^ mice developed severe acute inflammation throughout the lung parenchyma (Fig. 1A) when compared with their *Fra-1^+/+^* counterparts, which displayed a lesser degree of lung inflammation. As shown in Fig. 1B, the total number of cells recovered from the brochoalveolar lavage fluid (BAL) of the wild-type mice was approximately half that of the *Fra-1* mutant mice. The lung inflammation in *Fra-1* ^Δ/Δ^ mice was primarily characterized by greater accumulation of neutrophils and lymphocytes than in *Fra-1^+/+^* mice, which had only a few neutrophils and lymphocytes.

Given that *Fra-1* ^Δ/Δ^ mice showed greater levels of lung inflammation after bleomycin treatment, it was of interest to determine if similar changes in the expression levels of pro-inflammatory cytokines and chemokines were attributable to this response. Proinflammatory cytokines *TNF-α* and *IL-6* and chemokines, such as *MIP-1α*, *MIP-2* and *KC* mRNA expression were examined at day 7 after bleomycin treatment. The results revealed that *TNF-α* and *IL-6* mRNA levels were increased significantly on day 7 after treatment compared to sham controls (Fig. 1C). Though no difference in the expression of *IL-6* was observed between the two genotypes, *Fra-1* ^Δ/Δ^ mice showed a significant increase in *TNF-α* expression as compared with the wild-type mice upon bleomycin treatment on day 7. Bleomycin-induced *MIP-1α* and *MIP-2* expression was markedly increased on day 7 in *Fra-1* ^Δ/Δ^ mice as compared with their wild-type counterparts, whereas no difference was observed for the expression levels of *KC* between the genotypes (Fig. 1C). Taken together, these results suggest that Fra-1 may dampen bleomycin-induced lung neutrophilic inflammation *in vivo* at least, in part, by selectively suppressing the expression levels of pro-inflammatory cytokines and chemokines.

### Fra-1 Deficiency Promotes Lung Fibrosis at Day 14 of Post-bleomycin Administration

**Figure 2:**
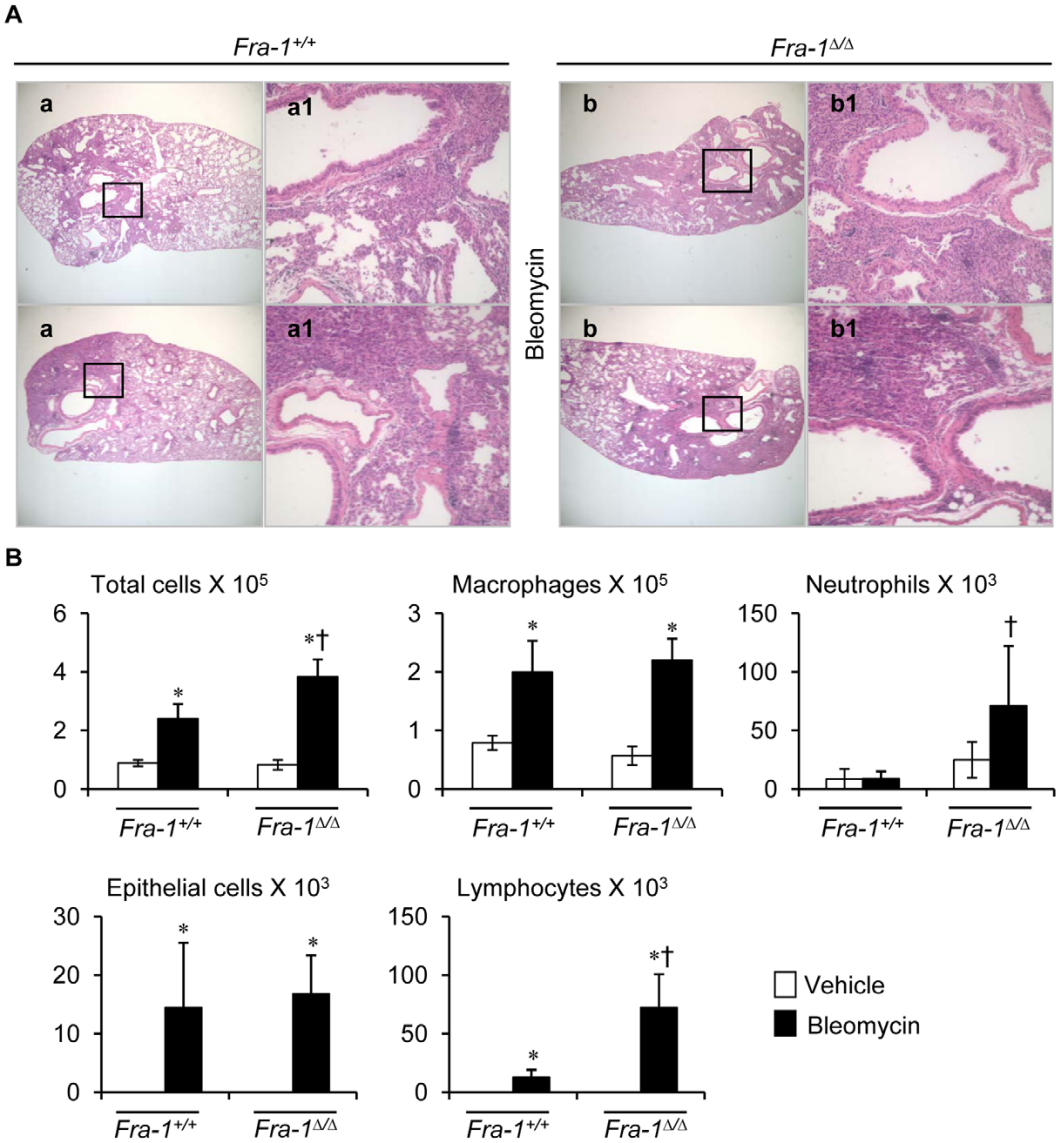
*Fra-1* ^Δ*/*Δ^ mice show extensive pulmonary inflammation and fibrosis following 14 days post-bleomycin instillation. Mice (n = 8 in each genotype) were intratracheally administered with bleomycin. Left lungs were fixed for histology (n = 4) or used for mRNA analysis (n = 4). Right lobes (n = 8) were used for BAL cell and protein analysis. Representative results of two different experiments are shown. Samples obatined from mice treated with PBS were from Figure 3 (see below). **A:** Representative images of H&E stained lung tissue sections of bleomycin-treated *Fra-1^+/+^* and *Fra-1* ^Δ*/*Δ^ mice (n = 4). **B:** Lung inflammatory cell profiles in BAL fluid of both genotypes (n = 4). ^∗^p*<* 0.05, PBS vs bleomycin; ^†^p*<*0.05, *Fra-1* ^Δ*/*Δ^ vs *Fra-1^+/+^* mice. Images **a** and **b** are at same magnification, ×4; **a1** and **b1** are at ×20 magnifications of **a** and **b,** respectively (boxed areas).

The representative lung histology shown at 14 days post-bleomycin instillation also revealed that *Fra-1* ^Δ/Δ^ mice had more severe inflammation, fibrosis, and alveolar wall thickening than did their wild-type counterparts (Fig. 2A). As shown in Fig. 2B, the total number of cells recovered from BAL fluids of the wild-type mice was approximately half that of the *Fra-1* mutant mice. The lung inflammation in *Fra-1* ^Δ/Δ^ mice was primarily characterized by significant accumulation of neutrophils and lymphocytes than in *Fra-1^+/+^* mice after bleomycin treatment. Despite there was no significant differences in macrophages and epithelial cells between the two genotypes, the absolute number of macrophages and epithelial cells was increased in both genotypes after bleomycin injury compared to their corresponding controls.

### Fra-1 Abrogation Compromises Late Fibrotic Responses Induced by Bleomycin *in vivo*

**Figure 3:**
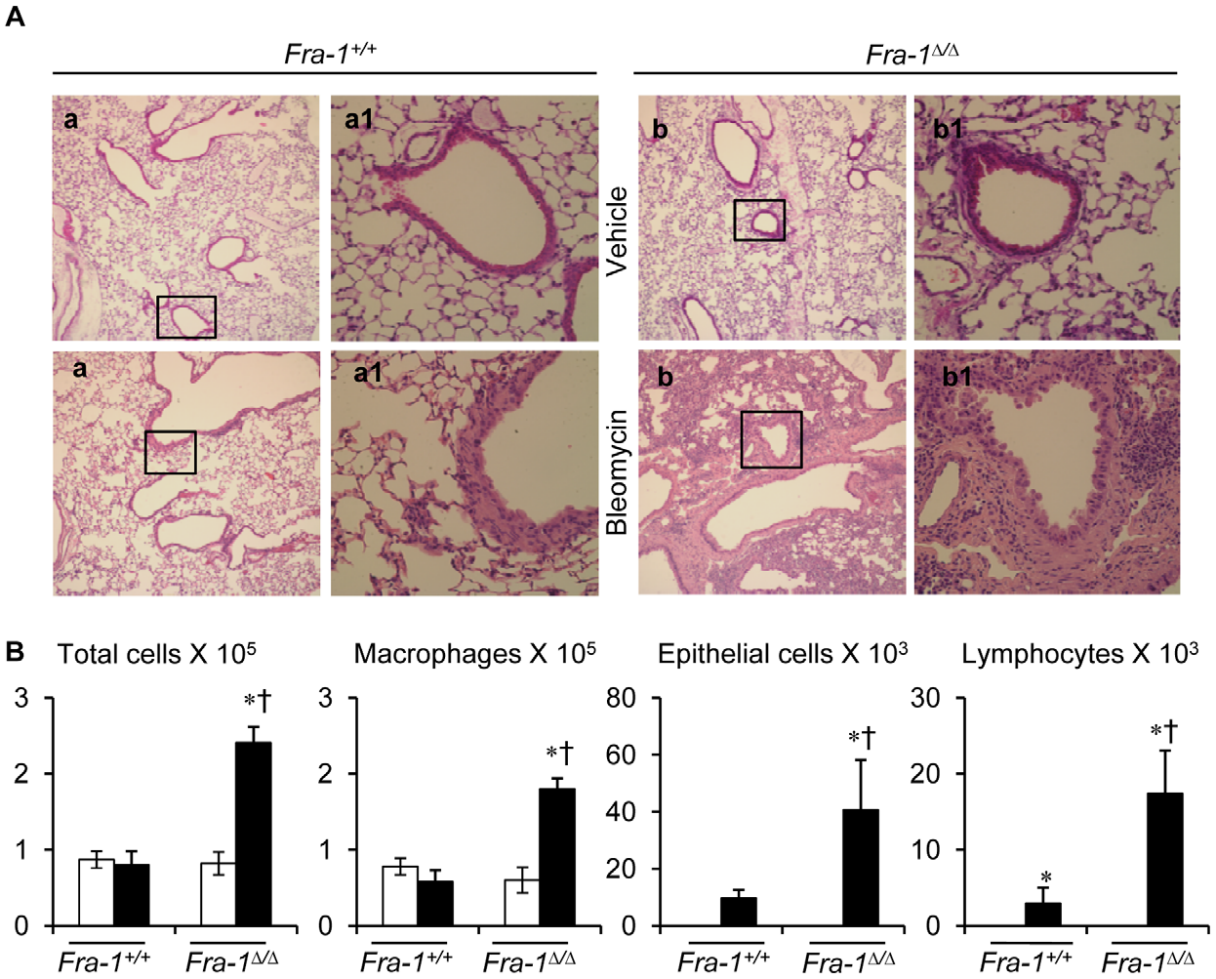
*Fra-1* ^Δ*/*Δ^ mice demonstrate extensive pulmonary inflammation and fibrosis at 31-day post-bleomycin administration. Mice were intratracheally administered with bleomycin (n = 14−15) or PBS (n = 12) were assessed. Representative results of three different experiments are shown. **A:** Representative images of H&E stained lung tissue sections of bleomycin-treated *Fra-1^+/+^* and *Fra-1* ^Δ*/*Δ^ mice (n = 4). **B:** Lung inflammatory cell profiles in BAL fluids at 31-day post-PBS and -bleomycin instillation (n = 5). Open bars  =  vehicle; filled bars  =  bleomycin. ^∗^p<0.05, PBS vs bleomycin; ^†^p<0.05, *Fra-1* ^Δ*/*Δ^ vs *Fra-1^+/+^* mice. Images in **a** and **b** are shown at x10, whereas **a1** and **b1** represent boxed areas of **a** and **b**, respectively, shown at x40.

We next investigated whether increased inflammation and fibrosis seen in *Fra-1* ^Δ/Δ^ mice persist by the end of 31 days. We found that, at the end of 31 days post-bleomycin treatment, *Fra-1* ^Δ/Δ^ mice displayed extensive inflammatory cell infiltration, granulomas in the perivascular region, inter-alveolar thickening of the septa, and a reduction in the alveolar space, as well as fibrosis. However, a modest degree of interstitial fibrogenesis and cellular infiltration were observed in *Fra-1^+/+^* mice, suggesting the Fra-1 is required for the resolution of lung fibrosis (Fig. 3A). Whereas, normal PBS treated lungs from *Fra-1* ^Δ/Δ^ did not display any differences when compared with their wild-type counterparts (Fig. 3A). Cell profile analysis of the BAL fluid also revealed significantly higher levels of macrophagic inflammation in the *Fra-1* ^Δ/Δ^ mice than in the *Fra-1^+/+^* mice (Fig. 3B). The expression levels of *TNF-α*, *IL-6, MIP-1α*, *MIP-2* and *KC* chemokines returned to basal levels and were not significantly different from sham controls of respective genotypes (data not shown).

### Fra-1 Deficiency Exaggerates Bleomycin-induced Lung Fibrosis

**Figure 4:**
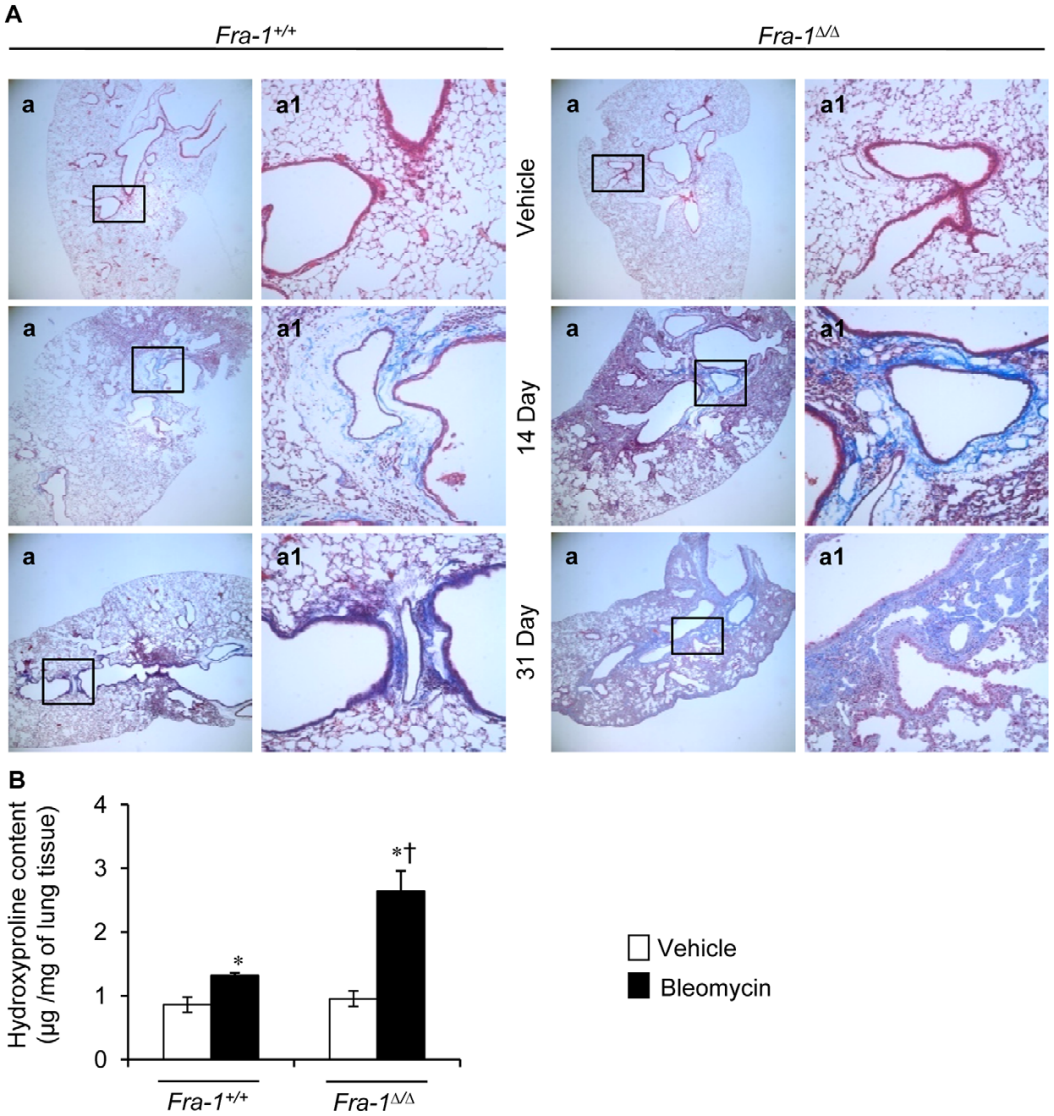
*Fra-1* ^Δ*/*Δ^ mice develop exaggerated pulmonary fibrosis after injury. A: Representative results of Masson’s trichrome staining of the lung from the saline-treated mice (n = 3) or bleomycin treated mice for 14 (n = 3) and 31 (n = 4) days. **B:** Right lung was collected for biochemical analysis of bleomycin-induced pulmonary fibrosis as measured by hydroxyproline content at 31-day post-PBS and -bleomycin treatment (n = 5). ^∗^p<0.05, PBS vs bleomycin; ^†^p<0,05, *Fra-1* ^Δ*/*Δ^ vs *Fra-1^+/+^* mice. Images in **a** are shown at x4, whereas **a1** represent boxed areas of **a**, shown at x20.

The increased extracellular collagen accumulation is a key abnormal event in fibrosis. We therefore next analyzed the extent of collagen deposition in lung tissues of *Fra-1* ^Δ/Δ^ and *Fra-1^+/+^* mice treated with vehicle or bleomycin. As shown by Masson’s trichrome staining (Fig. 4A), the lungs of mice examined 14 days after challenge typically demonstrated that *Fra-1* ^Δ/Δ^ mice had more peribronchiolar and parenchymal fibrosis than corresponding wild-type littermates. The extent of these changes at 14 days after bleomycin administration was substantially increased in *Fra-1* ^Δ/Δ^ mice at the end of 31 days. In contrast, the areas of collagen accumulation in the lungs of bleomycin-treated *Fra-1^+/+^* mice were fewer in number and considerably less dense at the end of 31 days. The lungs of PBS-treated mice appeared normal, with no collagen accumulation adjacent to large vessels or airways. Furthermore, to quantitatively assess the difference in the extent of fibrosis, we measured hydroxyproline as a surrogate marker for collagen deposition. Hydroxyproline levels were significantly lower in the lungs of *Fra-1^+/+^* mice than in *Fra-1* ^Δ/Δ^ mice treated with bleomycin for 31 days (Fig. 4B). Treatment of mice with PBS did not affect the lung hydroxyproline content, regardless of their genotype. Consistent with the morphological findings, these results indicate that the loss of Fra-1 results in increased levels of interstitial collagen deposition in response to bleomycin treatment.

### Fra-1 Deficiency Promotes Increased Levels of Pro-fibrotic and Fibrotic Gene Expression and Causes Altered Expression of MMPs and TIMPs *in vivo*

**Figure 5:**
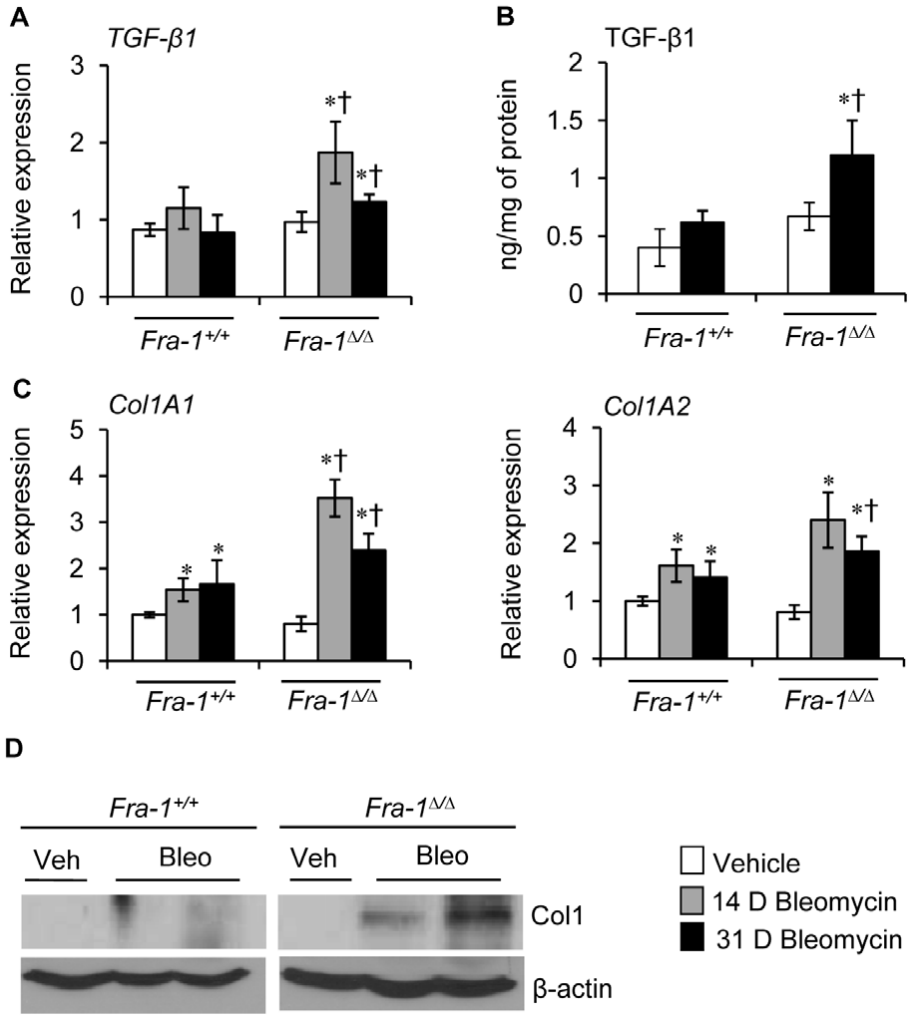
*Fra-1* ^Δ*/*Δ^ mice exhibit deregulated profibrotic and fibrotic gene expression following bleomycin instillation. Transcript expression was compared with 31 days PBS control mice. **A:** *TGF-β1* expression in the lungs of mice treated with PBS or bleomycin as analyzed by real-time RT-PCR (n = 4−6). **B:** TGF-β1 expression at day 31 following PBS or bleomycin instillation as detected by ELISA (n = 5). **C:** *Col1A1* and *Col1A2* expression as detected by real-time RT-PCR (n = 4−6). **D:** Col1 and β-actin expression at day 31 following bleomycin instillation as detected by immunoblot. ^∗^p < 0.05, PBS vs bleomycin; ^†^p < 0,05, *Fra-1* ^Δ*/*Δ^ vs *Fra-1^+/+^* mice.

**Figure 6:**
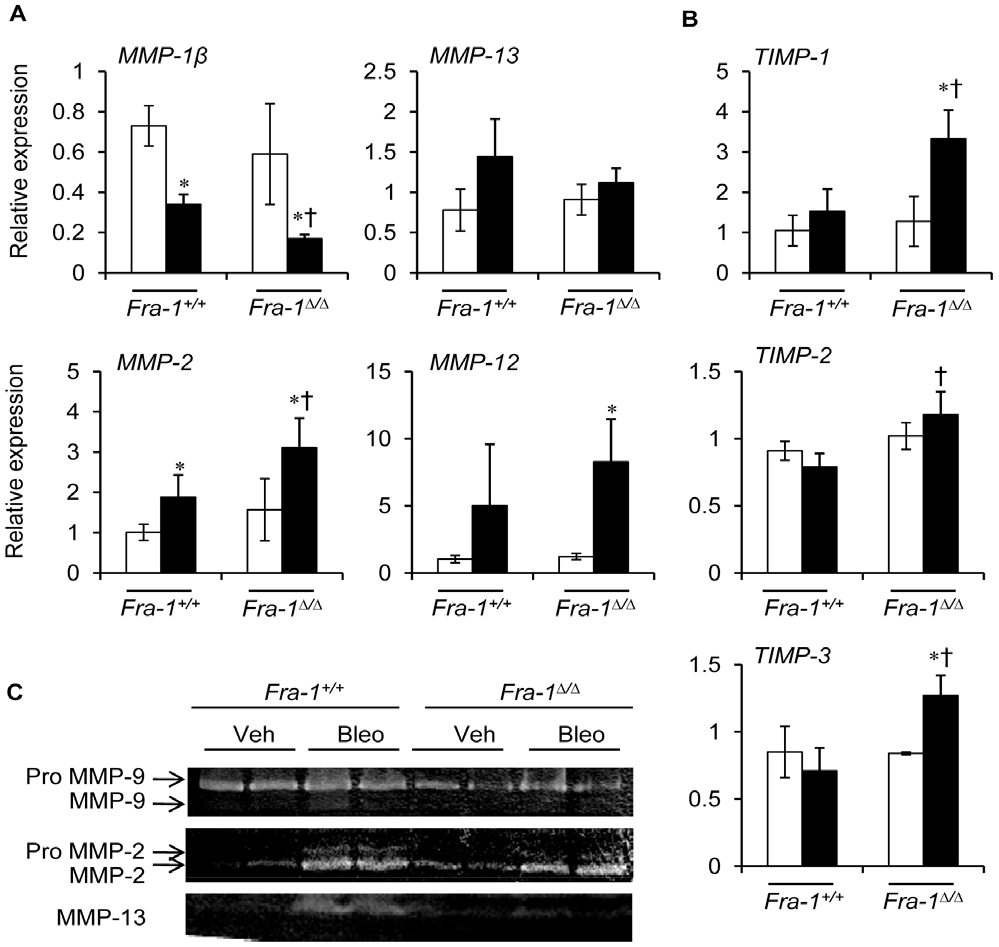
Loss of Fra-1 leads to an altered MMP and TIMP gene expression in the lung. *MMP* (**A**) and *TIMP* (**B**) gene expression in the lung tissue at 31 days post-bleomycin administration (n = 4−6). **C:** Detection of MMPs activity by collagen zymography in lung tissue 31 days post-bleomycin administration. Open bars  =  vehicle; filled bars  =  bleomycin. ^∗^p<0.05, PBS vs bleomycin; ^†^p<0,05, *Fra-1* ^Δ*/*Δ^ vs *Fra-1^+/+^* at corresponding time point.

Because the level of collagen accumulation in *Fra-1* ^Δ/Δ^ mice was higher than that observed in *Fra-1^+/+^* mice following bleomycin administration, we next asked whether these changes coincided with the increased expression of profibrotic and fibrotic genes, such as TGF-β1 and type 1 collagen (Col1), in the lungs of *Fra-1^+/+^* and *Fra-1* ^Δ/Δ^ mice. Real-time RT-PCR analysis revealed that bleomycin treatment at 14 and 31 (Fig. 5A) days significantly increased the expression levels of *TGF-β1* in *Fra-1* ^Δ/Δ^ mice when compared with those in *Fra-1^+/+^* mice. In agreement with the mRNA expression data, ELISA assays revealed significantly increased levels of TGF-β1 in the lungs of *Fra-1* ^Δ/Δ^ mice subjected to bleomycin for 31 days (Fig. 5B). In contrast, the level of this cytokine in *Fra-1^+/+^* mice treated with bleomycin was comparable to that found in saline-treated mice. The level of *Col1A1* expression was greater in bleomycin-treated *Fra-1* ^Δ/Δ^ mice than in *Fra-1^+/+^* mice at 14 and 31 days (Fig. 5C). Although no significant differences observed at the end of 14 days, the expression level of *Col1A2* was significantly higher in bleomycin-treated *Fra-1* ^Δ/Δ^ mice than in *Fra-1^+/+^* mice at 31 days (Fig. 5C). Immunoblot analysis using antibodies specific for Col1 revealed enhanced levels of Col1 in *Fra-1* ^Δ/Δ^ mice treated with bleomycin when compared with their corresponding vehicle control and *Fra-1^+/+^* counterparts (Fig. 5D). The accumulation of collagen depends not only on its production, but also on its degradation. Hence, we next analyzed the expression patterns of collagenases (*MMP-1β* and *MMP-13*), metalloelastase (*MMP-12*), and gelatinases (*MMP-2*). There was a significant reduction in *MMP-1β* transcript levels in the lungs of *Fra-1* ^Δ/Δ^ mice treated with bleomycin for 31 days (Fig. 6A) when compared with their wild-type counterparts. In contrast, the expression level of *MMP-13* was unaltered between two genotypes after bleomycin treatment (Fig. 6A). The expression levels of the gelatinase *MMP-2* and of the metalloelastase (*MMP-12*) were significantly different between the *Fra-1* ^Δ/Δ^ and *Fra-1^+/+^* mice subjected to 31 days of bleomycin. *MMP-2* expression was markedly up-regulated in *Fra-1* ^Δ/Δ^ mice when compared with *Fra-1^+/+^* mice. Although bleomycin-induced *MMP-12* expression was higher in the lungs of *Fra-1* ^Δ/Δ^ mice than *Fra-1^+/+^* mice, no significant difference in *MMP-12* induction was observed between the two genotypes. TIMP-1, TIMP-2, and TIMP-3 enzymes regulate MMP activity and ECM remodeling. mRNA expression analysis revealed elevated levels of these MMP inhibitors in the bleomycin-treated mice than in their *Fra-1^+/+^* counterparts (Fig. 6B). We then performed protein analyses of MMPs to confirm their abnormal mRNA expression in Fra-1 mutant mice after bleomycin treatment using collagen zymogram analysis. As shown in Figure 6C, MMP-2 activity was increased in the lungs of bleomycin-injured *Fra-1* ^Δ/Δ^ and *Fra-1^+/+^* mice when compared with the corresponding vehicle-treated controls. However, the activity of proMMP-2 was higher in *Fra-1^+/+^* mice than in their *Fra-1* ^Δ/Δ^ counterparts after bleomycin treatment. The activity of proMMP-9 was significantly higher in *Fra-1^+/+^* mice than in *Fra-1* ^Δ/Δ^ mice after bleomycin treatment, although the activity of MMP-9 was undetectable in both tissues. Bleomycin-induced MMP-13 activity was higher in *Fra-1^+/+^* mice than in PBS-treated animals. In contrast, undectable or low levels of MMP-13 activity were observed in *Fra-1* ^Δ/Δ^ mice. These data suggest that the elevated production of TGF-β1 and collagen induced by bleomycin and deregulated MMP and TIMP gene expression could contribute, in part, to the increased level of lung fibrosis in the Fra-1 mutant mice.

### Bleomycin Induces Fra-1 Expression in the Lung

**Figure 7:**
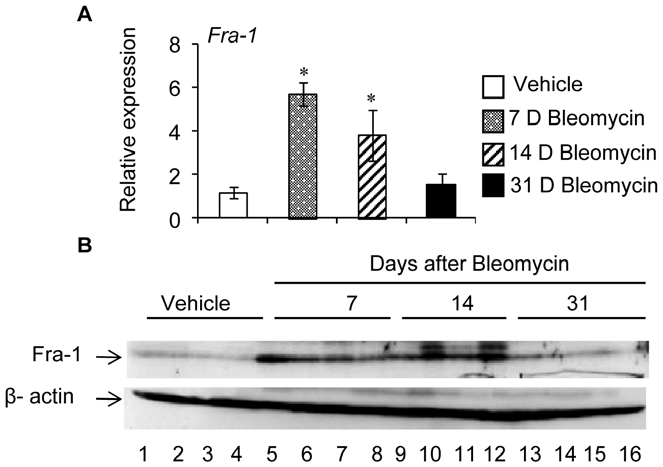
Bleomycin-induced Fra-1 expression in the lung. A: At indicated period of post-bleomycin instillation, lung tissues were harvested from wildtype mice and used for *Fra-1* gene expression by real-time RT-PCR (n = 4−6). **B:** Fra-1 expression was analyzed by western blot. analysis using β-actin as reference. Only 4 samples were used in each group. ^∗^p<0.05, PBS vs bleomycin treated groups.

To determine the nature of Fra-1 activation during bleomycin induced lung injury, we harvested lung tissues from *Fra-1^+/+^* mice with bleomycin for 7, 14, or 31 days post-instillation for RNA and protein analysis. Both real-time RT-PCR (Fig. 7A) and immunoblot analysis (Fig. 7B) demonstrated an induction of Fra-1 expression by bleomycin after 7 and 14 days, but not in the lungs of mice at 31 days post-bleomycin treatment.

### Fra-1 Knockdown in Human Lung Epithelial Cells Results in Greater Levels of Bleomycin-induced Fibrotic Response *in vitro*

**Figure 8:**
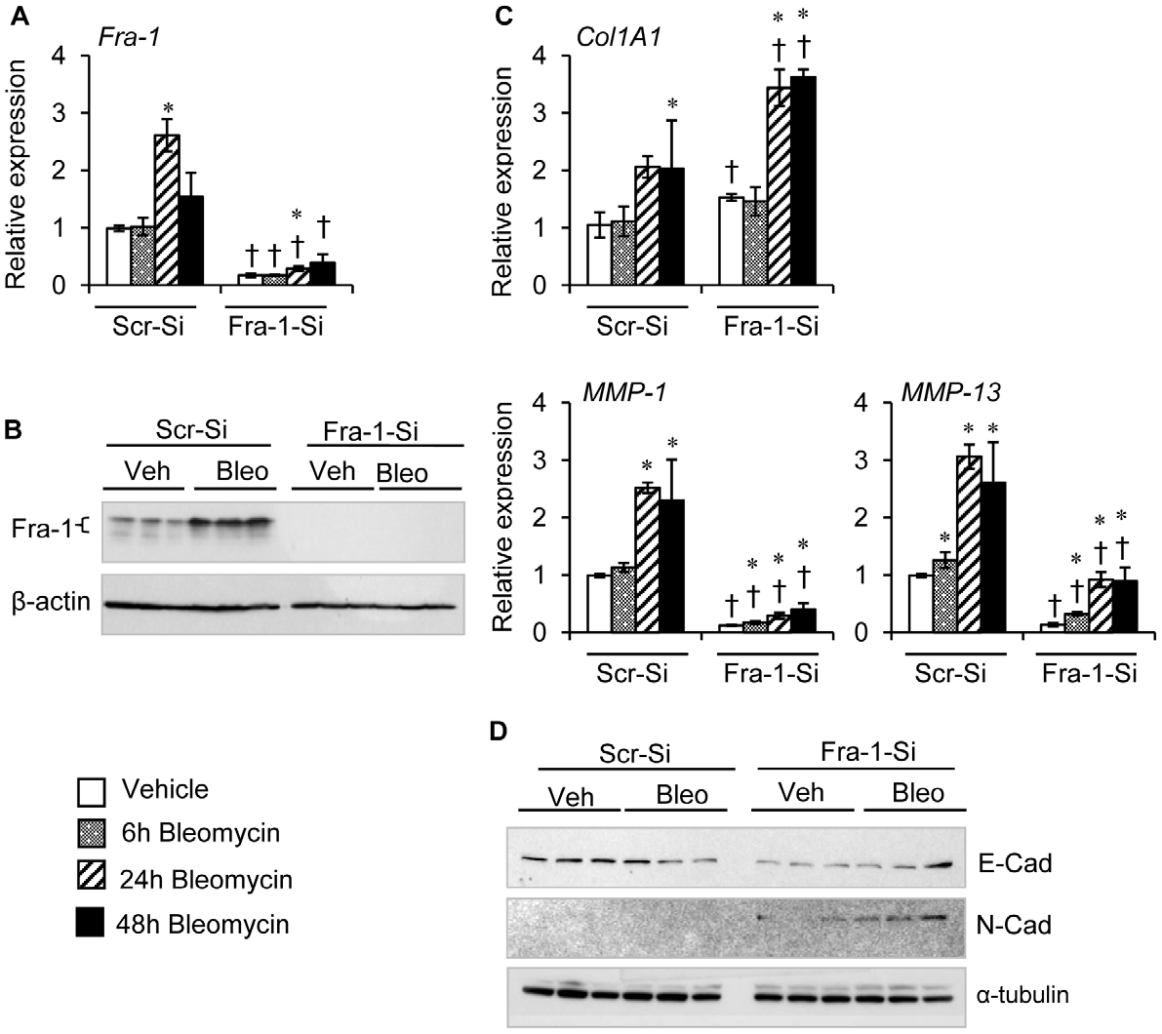
The effects of Fra-1 knockdown on bleomycin-induced mesenchymal marker, and fibrotic and anti-fibrotic gene expression in AECs. Cells were treated with vehicle or bleomycin (5 mU/ml) for different time points, and harvested for RNA and protein isolation. **A:** Expression of *Fra-1* analyzed by qRT-PCR. **B:** Fra-1 expression was analyzed by western blot analysis using β-actin as reference. **C:** *Col1A1*, *MMP-1* and *MMP-13* expression analyzed by qRT-PCR. **D:** E-cad, N-Cad, and α-tubulin detected by immunoblot in AECs treated with vehicle or bleomycin for 24 hours. Results are mean ± SD, representative of 2 separate experiments performed in triplicate per group. ^∗^P<0.05, PBS vs bleomycin; ^†^P *<* 0,05, Scr-si-RNA vs Fra-1-SiRNA cells at corresponding time points.

As epithelial cells are actively involved in the progression of fibrosis, we next examined the effects of Fra-1-deficiency on genes involved in morphological changes and bleomycin-induced fibrotic gene expression in human non-malignant small airway epithelial cells (AECs) immortalized by telomerase [21]. To examine the role of Fra-1, AECs were transfected with control (scrambled)-siRNA (hereafter refered to as Scr-si-AECs) or *Fra-1* siRNA (Fra1-si-AECs). To ascertain effciency of Fra-1 knockdown, RNA and protein was isolated from Scr-si-AECs and Fra1-si-AECs treated with and without bleomycin, and Fra-1 expression was quantified by real-time PCR and western analysis. Scr-si-AECs showed a significant increase in *Fra-1* expression after 24 hours of bleomycin treatment when compared with vehicle control. In contrast, Fra1-si-AECs showed a significantly lower level of *Fra-1* expression in control and bleomycin-treated groups (Fig. 8A). These results were further confirmed by western blot analysis; Scr-si-AECs revealed an increase in Fra-1 when compared with their vehicle-treated control and Fra1-si-AECs counterparts (Fig. 8B). To determine whether Fra-1 knockdown altered bleomycin-induced collagen expression, we analyzed *Col1A1* expression (Fig. 8C). Fra-1 knockdown markedly increased *Col1A1* expression under basal conditions. Upon bleomycin treatment, the expression level of *Col1A1* was markedly increased in Fra1-si-AECs, as compared with Scr-si-AECs. We next examined expression levels of collagenases (MMP-1 and MMP-13), which are known to regulate collagen turnover. As depicted in Fig. 8C, the transcript levels of MMP-1 and MMP-13 were markedly lower in Fra-1-si-AECs at basal level and even after bleomycin treatment when compared with corresponding Scr-si-AECs. Finally, we analyzed E-cadherin and N-cadherin to confirm their abnormal phenotype in Fra1-si-AECs (Fig. 8D) after 24 hours of bleomycin treatment. The expression of E-cadherin did not alter in Scr-si-AECs, but Fra-1 knockdown cells showed decreased expression levels of E-cadherin in both basal and treatment conditions. In contrast, Fra-1 knockdown caused an up-regulation in N-cadherin expression (mesenchymal marker), and this expression was persistently elevated even at the end of the 24 hour treatment as compared with Scr-si-AECs. Collectively, these results suggest that the loss of Fra-1 leads to an upregulation of fibrotic mediators, and also promotes the gene expression involved in an epithelial-mesenchymal transition.

### Fra-1 Knockdown in Human Lung Epithelial Cells Results in Greater Levels of TGF-β1-Induced Fibrotic Response *in vitro*

**Figure 9:**
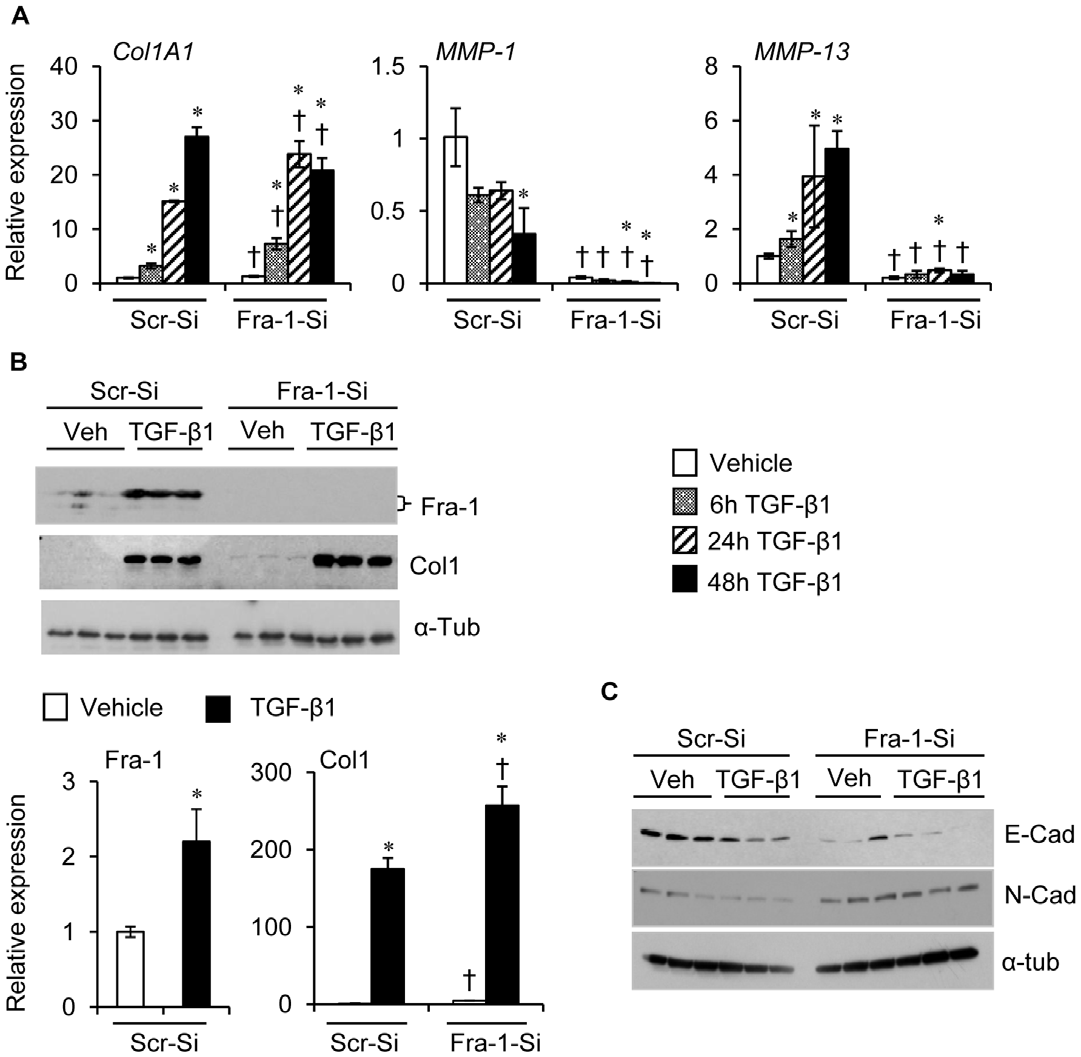
The effects of Fra-1 knockdown on TGF-β1-induced fibrotic and anti-fibrotic, and mesenchymal marker gene expression in AECs. Cells were treated with vehicle or TGF-β1 (5 ng/ml) for different time points, and harvested for RNA and protein isolation. **A:** *Col1A1*, *MMP-1* and *MMP-13* expression analyzed by qRT-PCR. **B:** Fra-1, Col1 and α-tubulin protein expression as analyzed by immunoblot in AECs cells treated with vehicle or TGF-β1 for 24 hours. **C:** E-cad, N-Cad, and α-tubulin detected by immunoblot in AECs treated with vehicle or TGF-β1 for 24 hours. Results are mean ± SD, representative of 2 separate experiments performed in triplicate per group. ^∗^P<0.05, PBS vs TGF-β1; ^†^P *<* 0,05, Scr-si-RNA vs Fra-1-SiRNA cells at corresponding time points.

It has been demonstrated that bleomycin induced fibrotic responses are mediated through TGF-β1 [28]. Also increased levels of TGF-β1 has been observed in the lung of *Fra-1* ^Δ/Δ^ mice treated with bleomycin in our study. Therefore, we sought to determine whether TGF-β1 was able to induce genes involved in morphological changes and fibrosis in the absence of Fra-1. A significant up-regulation of *Col1A1* expression was observed in Fra1-si-AECs at basal level and in response to TGF-β1, as compared with Scr-si-AECs (Fig. 9A). Analysis of MMPs expression revealed reduced levels of *MMP-1* and *MMP-13* in vehicle- and TGF-β1-treated Fra1-si-AECs compared with corresponding wild-type controls (Fig. 9A). TGF-β1 caused stimulation of Fra-1 expression in Scr-Si-AECs, but not in Fra1-si-AECs (Fig. 9B). In accordance with mRNA expression, immunoblot analysis also revealed increased levels of Col1 expression in both cell types. However, a significant difference was observed between them (Fig. 9B). The expression level of E-cadherin was significantly lower in Fra1-Si-AECs, and this level was further decreased by TGF-β1 treatment, as compared with their Scr-Si-AEC counterparts. In agreement with E-cadherin expression, an elevated N-cadherin expression was observed in the vehicle- and TGF-β1-treated Fra1-si-AECs compared with the Scr-si-AECs (Fig. 9C). The results of TGF-β1 treatment further support our hypothesis that Fra-1 regulates gene expression involved in both EMT and ECM remodeling.

### Fra-1 Knockdown Caused Altered Expression of Fibrotic and Anti-fibrotic Genes in A549 Cells Following Bleomycin and TGF-β1 Treatment

**Figure 10:**
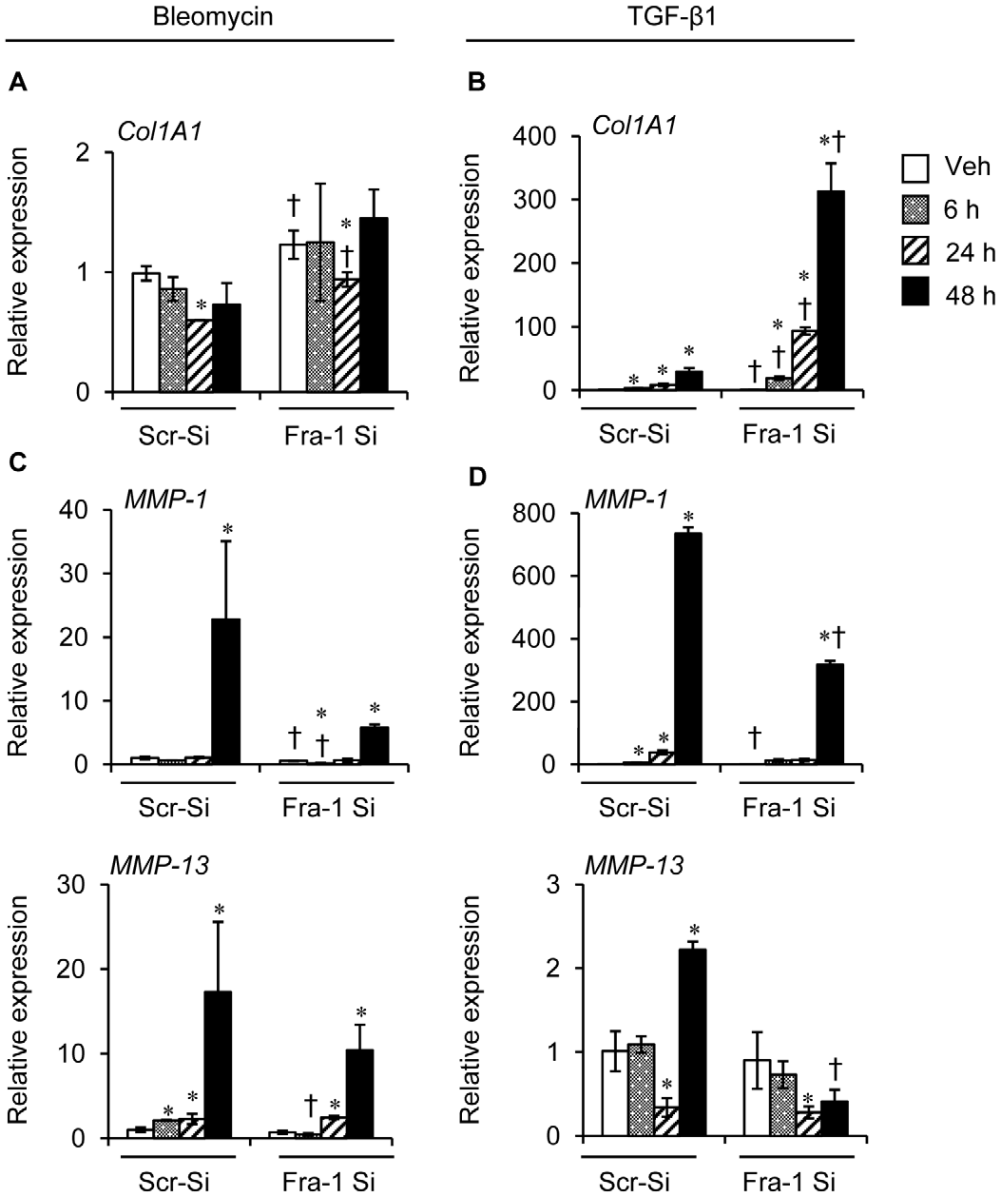
The effects of Fra-1 knockdown on bleomycin- or TGF-β1-induced fibrotic and anti-fibrotic gene expression in A549 cells. Cells were treated with vehicle or bleomycin (5 ng/ml) or TGF-β1 (5 ng/ml) for different time points, and harvested for RNA isolation. *Col1A1* mRNA expression induced by bleomycin (**A**) and TGF-β1 (**B**). *MMP-1 and MMP-13 *mRNA expression induced by bleomycin (**C**) and TGF-β1 (**D**). Results are mean ± SD, representative of 2 separate experiments performed in triplicate per group. ^∗^P<0.05, PBS vs bleomycin or TGF-β1; ^†^P *<* 0,05, Scr-si-RNA vs Fra-1-SiRNA cells at corresponding time points.

Further, to verify the effects of Fra-1 deficiency on fibrotic and anti-fibrotic gene expression induced by pro-fibrogenic agents, we performed selective experiments on a human malignant lung type-II like epithelial cell line, A549. These cells were transfected with a control (scrambled)-siRNA or a *Fra-1* siRNA, and the effciency of Fra-1 knockdown was evaluated by real-time PCR analysis (data not shown). As shown in Fig. 10A, Fra-1 knockdown caused a significant increase in the expression levels of *Col1A1,* as compared to scrambled-siRNA transfected cells, under basal conditions. In contrast to bleomycin, which did not markedly alter the expression levels of collagen (Fig. 10A), TGF-β1 treatment caused the stimulation of *Col1A1* expression in A549 cells with the Fra-1 knockdown and the magnitude of induction was markedly higher than that noted in A549 cells without the Fra-1 knockdown (Fig. 10B). As shown above, Fra-1 knockdown resulted in the downregulation of bleomycin- and TGF-β1-induced *MMP-1 and MMP-13* expression in non-malignant lung epithelial cells (Figs. 8 and 9). Thus, we have examined the expression patterns of these two MMPs in A549 cells with and without the Fra-1 knockdown after bleomycin and TGF-β1 treatment. Bleomycin induced *MMP-1 and MMP-13* expression was significantly lower in A549 cells transfected with *Fra-1* siRNA, as compared with their A549 counterparts transfected with the scrambled-siRNA (Fig.10C). Likewise, *MMP-1* and *MMP-13* expression induced by TGF-β1 was markedly lower in A549 cells transfected *with Fra-1* siRNA, as compared with control siRNA (Fig. 10D).

## Discussion

Our results demonstrate that Fra-1-deficient mice develop a severe and progressive lung fibrosis, suggesting that Fra-1 negatively regulates the development of fibrosis. Our findings related to the initial inflammatory events, and then late fibrotic responses, in the *Fra-1* ^Δ/Δ^ mice in response to bleomycin have important implications about the role of Fra-1 in the development of lung fibrosis *in vivo*. The greater neutrophilic alveolitis seen in the lungs of *Fra-1* ^Δ/Δ^ mice at day 7 of the post-bleomycin challenge were associated with a transient up-regulation of cytokine and chemokine gene expression such as *TNF-α*, *MIP-1α* and *MIP-2* (Fig. 1C). Previous studies have shown that excessive production of these proinflammatory molecules contribute to fibrosis, as administration of antagonists for these gene products attenuated experimental lung fibrosis *in vivo* [29], [30], [31]. In experimental models of lung fibrosis induced by silica or bleomycin, the increase in neutrophil number and the duration of tissue neutrophil activation have also been found to be correlated with chronic alveolitis progressing to fibrosis [32], [33]. Furthermore, neutrophil migration and activation are usually higher in patients with a fibrotic lung disorder that is more aggressive and has a worse prognosis [34], [35]. In this study, we found that increased severity of bleomycin-induced fibrosis in Fra-1-deficient mice coincided with a significantly increased level of early neutrophilic inflammation at 7 days post-bleomycin, when compared with their wild-type counterparts. In the late stages of lung fibrosis development (after 31 days), *Fra-1* ^Δ/Δ^ mice developed a more severe and progressive interstitial fibrosis, with greater influx of lymphocytes and macrophages, than their wild-type counterparts. Other studies show a similar kind of lung cellular infiltration at early and late stages of bleomycin-induced fibrosis in rodents [36], [37]. Collectively, these results suggest that Fra-1 is important in modulating bleomycin-induced lung fibrosis *in vivo*, and this transcription factor exerts its effects by dampening pro-inflammatory cytokine and chemokine gene expression.

Alternatively, tissue fibrosis can occur when collagen production outpaces collagen degradation, under both homeostatic conditions and in conditions of rapid matrix remodeling [6]. Thus, we asked whether bleomycin would cause changes in the expression of specific MMPs in *Fra-1* ^Δ/Δ^ mice, resulting in an enhanced severity of lung fibrosis. Our studies revealed that increased levels of collagen production after bleomycin-induced lung injury contributes to enhanced severity of lung fibrosis in *Fra-1* ^Δ/Δ^ mice. Moreover, we found that expression levels of several MMPs were dysregulated in the lungs of bleomycin-treated *Fra-1* ^Δ/Δ^ mice when compared with *Fra-1^+/+^* mice. MMP-2 gene expression was significantly elevated in the lungs of Fra-1-deficient mice at 31 days post-bleomycin treatment. However, MMP-2-deficient mice have been reported to exhibit a reduced level of inflammation in their airspaces when compared with wild-type mice in an asthma model of lung disease, indicating a negative role for this MMP in pulmonary inflammation [38]. Thus, it is likely that MMP-2 plays a less significant role in pulmonary fibrosis under our experimental conditions. In contrast to MMP-2, we have observed an up-regulation of MMP-13 expression and activity in the *Fra-1^+/+^* mice, but not in the *Fra-1* ^Δ/Δ^ mice, following bleomycin treatment (Fig. 6). The interstitial collagens (types I, II, and III) are the principal targets of destruction, and the secreted collagenases (MMP-1 and MMP-13) play a major role in this process [39]. Thus, we speculate that MMP-1 and MMP-13 activation may be a cellular response designed to degrade the excess collagen that has accumulated after the bleomycin injury in wild-type mice, but decreased levels of MMP-1 and MMP-13 up-regulation may also be one of the causes of collagen accumulation and fibrosis in *Fra-1* ^Δ/Δ^ mice. This notion is also supported by a recent report showing that MMP-13 during the initial phase of fibrogenesis, could be important for the cleavage of newly formed matrix in an experimental model of hepatic fibrosis [40]. Furthermore, increased serum levels of MMP-13 have been reported in conjunction with systemic sclerosis in humans, further supporting its relevance in fibrosis [41].

MMP activity is generally counterbalanced by the presence of TIMPs. TIMP-1 and TIMP-2 are expressed by a variety of cell types, and are known to play a major role in regulating pulmonary fibrosis [42], [43]. Increased levels of TIMP gene expession have been reported in the lungs of IPF patients [6]. Up-regulation of *TIMP* gene expression noted in the lungs of *Fra-1* ^Δ/Δ^ mice suggests that Fra-1 negatively regulates TIMP gene expression [44]. Alternatively, the increased levels of TIMP gene expression may be due to the presence of elevated levels of TGF-β1 observed in the lungs of *Fra-1* ^Δ/Δ^ mice treated with bleomycin (Fig. 5). Indeed, TGF-β1-mediated signaling has been shown to up-regulate TIMP gene expresssion in the lungs of mice with TGF-β1–induced lung fibrosis model [45]. Based on these observations, we propose that the loss of Fra-1 results in increased levels of TIMP gene expression, which may serve to inhibit MMPs responsible for degrading excess collagen, thereby resulting in an enhanced severity of lung fibrosis in *Fra-1* ^Δ/Δ^ mice.

Impaired epithelial-mesenchymal transition (EMT) and fibroblast transdifferentiation have been shown to contribute to the pathology of pulmonary fibrosis [46], [47]. Both the lung alveolar and bronchial epithelial cells undergo epithelial-mesenchymal transition when exposed to profibrotic agents [48], [49]. EMT not only occurs in fibrosis but also happens during cancer cell invasion and metastasis in multicellular organisms. Fra-1 can promote invasion and the transition of tumor cells from an epithelial to a mesenchymal morphology. For instance, Fra-1 regulates Ha-RAS-induced EMT in human colon carcinoma cells [50]. In other study, it was shown that Fra-1 induction drives EMT through the regulation of miR-221/222 expression in breast cancer cell lines [51]. Similarly, c-Fos induces EMT in mouse epithelioid carcinoma cells and this is associated with a decrease in E-cadherin expression [52]. Though published results suggest a regulatory role for Fra-1 in EMT in tumorigenesis, whether or not Fra-1 regulates EMT in non-malignant lung epithelial cells in response to pro-fibrotic agents are largely undefined. Therefore, to further strengthen the role of Fra-1 on specific lung cell types, these *in vivo* findings were extended to *in vitro* studies, whereby small airway epithelial cells and fibroblasts were used to provide a mechanistic explanation for the protective effects of Fra-1 on pulmonary fibrosis. We have noted a significant up-regulation of markers of EMT and a loss of E-cadherin expression in lung epithelial cells with Fra-1 knockdown (Fig. 8D). Likewise, Fra-1 deficiency caused the induction of transdifferentiation of human lung fibroblasts into myofibroblasts as revealed by α-smooth muscle actin expression (data not shown). These results suggest that Fra-1 negatively regulates EMT and myofibroblast transdifferentiation of non-maligant lung epithelial cells and fibroblasts, respectively, to dampen the lung fibrosis induced by profibrotic agents, such as bleomycin. However, the exact mechanism by which Fra-1 deficiency leads to the induction of EMT warrants further study.

In addition, treatment of lung epithelial cells with bleomycin or TGF-β1 resulted in an increased expression of *Col1A1* in Fra-1 knockdown cells, in a manner similar to that observed in the intact lung *in vivo* (Fig. 5). In contrast, we found diminished levels of *MMP-1* and *MMP-13* expression induced by bleomycin or TGF-β1 in lung epithelial cells transfected with Fra-1-siRNA, when compared with control-SiRNA transfected cells. Previous studies have shown that Fra-1 can directly activate the expression of several genes that regulate the ECM turnover in several cell types, including the lung epithelial cells [12]. Moreover, the presence of Fra-1 binding sites (AP-1 sites) in human MMPs and TIMPs, (e.g., MMP-1, MMP-9, MMP-13, TIMP-1 and TIMP-3) promoters have been reported [12], [13], [14], [15], [16]. The exact mechanisms by which the loss of Fra-1 results in altered expression levels of collagen and collagenases in lung epithelial cells remain to be established.

Recently, a role for AP-1 family members, including Fra-1, has been shown in both inflammatory and proliferative diseases. Overexpression and knockdown of various members of the AP-1 family under the influence of ubiquitous promoters have previously been described in mice [53], [54]. Overexpression of Jun-D causes lymphopenia [55], and its knockdown reduces hepatic fibrosis [56]. c-Fos has been reported to primarily target the bone, and mice overexpressing c-Fos develop osteosarcoma [57]. Eferl and coworkers demonstrated that transgenic expression of Fra-2, which is closely related to Fra-1, in mice causes lung fibrosis, characterized by vascular remodeling and fibrogenesis [58]. Recently, a supporting role for Fra-1/AP-1 signaling in the development of hepatic fibrosis has been described [59]. Mice expressing ectopic Fra-1 has been shown to spontaneously develop biliary fibrosis with increased levels of hepatic collagen content, accompanied by increased expression levels of profibrotic genes (e.g., *TGF-β1*, *MMP-2*, *MMP-9* and *TIMP-1*) [59]. Mice expressing ectopic Fra-1 has been shown to display impaired chondrogenesis during fracture healing, and this was associated with dimished expression levels of pro-inflammatory mediators, such as *TNF-α*, IL-6 and Cox-2 [60]. In a different study, it was shown that, in response to combined gefitinib (EGFR inhibitor) and LPS treatment, mice expressing ectopic Fra-1 develop interstitial lung disease, accompanied by diminished levels of *TNF-α*, *MIP-1α* and *MIP-2* when compared with wild-type mice [61]. In contrast, Fra-1-deficient mice are less susceptible than wild-type (Fra-1-sufficient mice) mice to LPS-induced lung injury and inflammation and death [61], [62]. The discrepancy observed between *Fra-1* ^Δ*/*Δ^ mice (which develop more fibrosis) and Fra-1 transgenic mice (which develop more hepatic fibrosis and interstitial lung disease) could be attributed to a different transcriptional response induced by pro-inflammatory and pro-fibrotic agents in the absence, or in the presence (overexpression), of Fra-1. Alternatively, this could be due to the differences in experimental (hepatic vs lung) and treatment (LPS +EGFR vs bleomycin) conditions. Nonetheless, these studies suggests that Fra-1 regulates inflammatory and pro-fibrotic responses in a context-dependent manner.

In conclusion, our results demonstrate that Fra-1 is required to limit tissue fibrosis *in vivo* after bleomycin injury. Moreover, *in vivo* and *in vitro* studies suggest that the protective effect of Fra-1 on pulmonary fibrosis is related to its role in regulation of pro-inflammatory events, pro-fibrotic and fibrotic gene expression and transdifferentiation of epithelial cells and fibroblasts. Although various effector pathways that either promote or limit the development of lung fibrosis have been reported, at present there is no effective therapy available for lung fibrosis. Thus, targeting (activating) the Fra-1/AP-1 pathway may provide a promising approach to treat lung fibrosis.
